# Development and Characterization of Novel Composite Films Based on Soy Protein Isolate and Oilseed Flours

**DOI:** 10.3390/molecules26123738

**Published:** 2021-06-19

**Authors:** Magdalena Mikus, Sabina Galus, Agnieszka Ciurzyńska, Monika Janowicz

**Affiliations:** Department of Food Engineering and Process Management, Institute of Food Sciences, Warsaw University of Life Sciences-SGGW, 159c Nowoursynowska St., 02-776 Warsaw, Poland; magdalenamikus1996@gmail.com (M.M.); agnieszka_ciurzynska@sggw.edu.pl (A.C.); monika_janowicz@sggw.edu.pl (M.J.)

**Keywords:** edible films, soy protein, oilseed flour, mechanical and barrier properties

## Abstract

The possibility of using oilseed flours as a waste source for film-forming materials with a combination of soy protein isolate in preparation of edible films was evaluated. Physical, mechanical and barrier properties were determined as a function of the oilseed type: hemp, evening primrose, flax, pumpkin, sesame and sunflower. It was observed that the addition of oilseed flours increased the refraction and thus the opacity of the obtained films from 1.27 to 9.57 A mm^−1^. Depending on the type of flours used, the edible films took on various colors. Lightness (L*) was lowest for the evening primrose film (L* = 34.91) and highest for the soy protein film (L* = 91.84). Parameter a* was lowest for the sunflower film (a* = −5.13) and highest for the flax film (a* = 13.62). Edible films made of pumpkin seed flour had the highest value of the b* color parameter (b* = 34.40), while films made of evening primrose flour had the lowest value (b* = 1.35). All analyzed films had relatively low mechanical resistance, with tensile strength from 0.60 to 3.09 MPa. Films made of flour containing the highest amount of protein, pumpkin and sesame, had the highest water vapor permeability, 2.41 and 2.70 × 10^−9^ g·m^−1^ s^−1^ Pa^−1^, respectively. All the edible films obtained had high water swelling values from 131.10 to 362.16%, and the microstructure of the films changed after adding the flour, from homogeneous and smooth to rough. All blended soy protein isolate–oilseed flour films showed lower thermal stability which was better observed at the first and second stages of thermogravimetric analysis when degradation occurred at lower temperatures. The oilseed flours blended with soy protein isolate show the possibility of using them in the development of biodegradable films which can find practical application in the food industry.

## 1. Introduction

Edible films and coatings obtained on the basis of biopolymers are bio-based packaging which can find applications as protective coatings or be consider as bioplastics used for food packaging. They are made of renewable and biodegradable materials that are suitable regarding environmental issues. Carbohydrates and proteins are commonly used in the production of films, but in order to improve their properties, additives of other ingredients, e.g., lipids, are used [1]. Today, edible films find many applications in the food industry because the application of a thin-film layer on or between food components enables an improvement in the quality of the product, as well as the extension of its shelf life when applied as a protective edible coating for fruits or vegetables. In addition, edible films protect food products against mechanical damage and protect the product against the adverse effects of physical, chemical and microbiological factors [2]. The most commonly used biopolymers for their synthesis are proteins, polysaccharides and lipids, and it is also possible to obtain composite films. The ingredients used in the film are mostly of plant origin, e.g., waxes, oils, zein or cellulose [3]. A frequently investigated ingredient for creating biodegradable films is soy protein isolate because it is an inexpensive, readily available and nutritious protein. Previous studies show that soy protein isolate has good film-forming properties, creating films with adequate mechanical and barrier properties. Edible films based on soy protein isolate are characterized by a significant smoothness and flexibility [4].

The hypothesis of this research was to use oilseed flours as film-forming materials in order to recycle these waste materials after the production of oils. Preliminary research showed that films obtained only from oilseed flours were brittle and did not form a continuous structure. Therefore, soy protein isolate, a similar plant-source polymer, was used as a film-forming material. Oilseeds are grains that contain a high fat content, which may exceed 40%. The growing interest in oilseeds in recent times results from a better understanding of their chemical composition, and the factors that influence it include environmental conditions, genetic conditions and conditions during the processing of raw materials [5]. The seeds obtained from oilseeds are also a valuable source of vitamins and essential fatty acids (EFA) [6]. In recent years, the use of processing residues from the fruit and vegetable industry has aroused great interest in the production of edible materials. They are increasingly used for the production of flours, which have a promising potential for the production of film-forming materials [7]. Flours are materials that have a lower shelf life than grains. This is due to damage to the seed coat during the production process [8]. Flours made from legumes can also be used as a film-forming material. In addition to being a good film-forming material, they are a good source of nutritional protein and also contain numerous vitamins and minerals [2]. To date, there has been little research conducted using oilseed flours to form edible films. On the other hand, research was carried out to obtain edible films from other types of flours, including eggplant flour and corn starch [9], quinoa [10] and chia seeds [11]. According to the obtained results, it was found that most of the obtained edible films were characterized by a heterogeneous structure and poor mechanical properties. Ochoa-Yepes, Medina-Jaramillo, Guz and Famá [12] investigated cassava starch films prepared with the addition of lentil flour, made from residues from the production of lentil protein. The addition of flour made the edible films characterized by a higher breaking strength and greater elasticity and flexibility. Another alternative source for the production of biodegradable films is achira flour. Films obtained from this flour were characterized by low solubility in water and relatively good barrier resistance against water vapor [13].

The aim of this study was to develop and characterize functional properties of edible films based on different types of oilseed flours and soy protein isolate. According to the authors’ knowledge, the types of flours used in this work were used for the first time as edible films, which would be an innovative “novelty” and an interesting means for commercial use as well as an alternative to the use of chemical treatments. The influence of adding flour on the thickness, water solubility, swelling index, optical properties (opacity and color), water vapor permeability, sorption kinetics, microstructure and mechanical and thermal properties of the films was analyzed. The size of the oilseed flour particles was also determined.

## 2. Results

### 2.1. Oilseed Flour Particle Size Measurement

**Figure 1:**
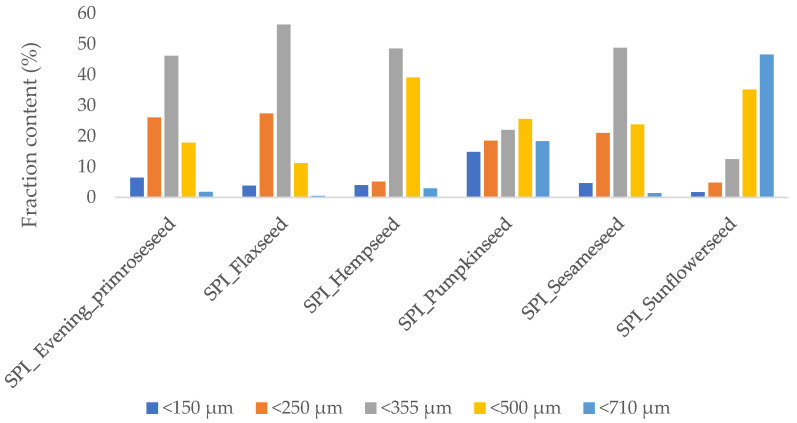
Fraction content in oilseed flours.

Flour is a heterogeneous mixture of particles of various sizes, densities and shapes. Figure 1 shows a diagram of the dependence of the size of the flour fraction on the share of individual fractions in a given type of oilseed flour (evening primrose, flax, hemp, pumpkin, sesame and sunflower). Detailed granulometric analysis showed that in all types of commercial flours used, the 355 and 500 μm fractions constituted the highest percentage. The highest value describing the content of the fraction of 710 μm was observed for sunflower flour, which was characterized by high cohesiveness. Pumpkin seed flour is characterized by a high content of iron and copper, while sesame flour is a rich source of calcium and has emulsifying properties [14]. Sesame protein isolate, which is used in food coatings, is obtained from sesame flour obtained after an extraction process to obtain oil [15]. According to Patwa, Malcolm, Wilson and Ambrose [16], the flour particle size is an important quality parameter because the size of the particles affects the type of processing technique used and generates the final product quality. Due to the simplicity and ease of analysis in laboratory conditions, sieve analysis is a method often used in the industry. Those authors observed the geometric mean particle diameter for hard red and soft white wheat flours at 142.30 and 693.10 µm, respectively. This range, observed for 10 min of sieving time, is similar to the values obtained for the analyzed oilseed flours. Seeds characterized by greater hardness have a stronger bond between the starch and protein, which also results in larger flour particles. The observed differences in the content of individual fractions may depend on the heterogeneity of the chemical composition of the raw material and the type of flour production process. In order to improve fluidity and enable easier screening of the cohesiveness of flour, an appropriate flow-improving agent should be used, e.g., calcium phosphate. Additionally, according to Adjei-Fremah et al. [17], the particle size distribution is a very important factor determining the functional properties, particularly the hydration properties and the quality of developed product. The hydration properties are greatly influenced by porosity, as well as by chemical features, which include the number and distribution of polar functional groups that have the ability to bind water. Those authors obtained the mean diameter of the volume distribution for whole cowpea flours between 33.3 and 432.3 µm, which is lower than that for the oilseed flours presented in this paper. Preliminary studies were performed for various oilseed flours, but crushing of the edible films occurred due to the large particles. To reduce the brittleness of the edible films, flours with fractions below 250 μm were used for the preparation of films.

### 2.2. Physical Properties

#### 2.2.1. Water Content

The tested edible films had a water content of 6.20–8.50% (Table 1), which had a significant impact on the physical and barrier properties of the films obtained. The films with the highest sugar content, i.e., those obtained on the basis of sunflower or linseed flour, achieved the highest percentage of water content and the greatest tendency to swell in water (Table 1). Higher values of water content (15.08–17.73%) were obtained by Pająk, Przetaczek-Rożnowska and Juszczak [18], who tested edible films based on starch from pumpkin, lentils and quinoa. Those authors observed that for films made of corn starch, ahipa and cassava, and with the addition of 30% glycerol, the moisture content is much higher at 15.3, 19.8 and 26.3% than in the case of films with a lower glycerol content. Glycerol is a hydrophilic plasticizer that has water-holding properties. Moreover, the addition of a plasticizer reduces the interactions between starch macromolecules [19]. Additionally, Andrade-Mahecha, Tapia-Blácido and Menegalli [20] obtained a higher water content in edible films made of achira flour (18.2 ± 0.7%). The high water content of edible films may be due to the high amount of hydrophilic components, such as protein, carbohydrates and fiber, in the flour. The high content of these ingredients can cause interactions with water molecules, which means that more water is retained in the edible film [9].

#### 2.2.2. Thickness

Differences were observed in the edible films obtained, despite the use of the same amount of ingredients for the preparation of film-forming solutions (Table 1). The control film of soy protein isolate (120.5 ± 12.50 μm) and the film of sunflower flour (137.6 ± 14.25 μm) were characterized by the lowest thickness. The film made of evening primrose flour was characterized by the highest thickness value (173.5 ± 15.21 μm). The differences in thickness may have been due to the different densities of the prepared film-forming solutions, since viscous solutions tend to form thicker layers. In films obtained from pinhão flour by Daudt et al. [21], the thickness varied with total solids, from 0.039 mm for films without glycerol to 0.057 mm for films with 1.5% glycerol. The differences in the thickness of the homogeneous films did not significantly affect the water vapor permeability or the mechanical properties of the films. Chandla et al. [22] obtained amaranth, buckwheat and corn starch films with an average thickness of 0.24, 0.28 and 0.29 mm, characterized by a uniform and smooth surface.

#### 2.2.3. Water Solubility

The potential application of edible films may require a high water resistance to increase the product integrity and obtain a moisture-resistant package. In other instances, however, good solubility of the films prior to use may be desired, such as when encapsulating food additives [23]. Control films made of soy protein isolate were characterized by significant water solubility, similar to the study by Galus [4], while films with the addition of oilseed flour did not dissolve when placed in water (Table 1).

Aydogdu et al. [24], when analyzing the obtained lentil flour edible films, observed that the films retained their physical integrity. The solubility of the tested films with the addition of oilseed flour was quite low, and the lowest solubility value was found for the pumpkin seed flour film, which, compared to the composition of other flours used, contained the largest amount of fat. According to Basiak, Lenart and Debeaufort [25], the water solubility of starchy materials is related to the amylose content. Those authors obtained water solubility in the range 30.16–34.76%, which is much higher than that for the analyzed films here. The higher the amylose content of the flours used, the lower the water solubility index of the films. The differences in the obtained values may also result from the different thicknesses of the films and the inhomogeneous and porous structure. According to Gutiérrez [26], the presence of methyl groups derived from pumpkin flour in edible films reduces the number of hydroxyl groups that have the ability to remove moisture. In addition, nanocomposites incorporated into edible films may reduce their hydrophilicity.

#### 2.2.4. Swelling Index

The ability of the edible films to swell in water is an important parameter as it provides information about the water resistance of the tested packaging materials. It also helps maintain the quality of food products during storage. The prepared edible films showed a swelling parameter in the range of 131.10–362.16% (Table 1), while the film made of soy protein isolate lost its integrity and dissolved when placed in water. The highest value of swelling in water was obtained for the flaxseed flour edible film (362.16%), and the lowest was obtained for the pumpkin seed flour film (131.10%). The films of sesame flour and pumpkin seeds, which had the highest content of fat in their composition, showed a similar dependence of swelling after being placed in water. Edible films that tend to absorb a large amount of water can alter the appearance, texture, durability and taste of the coated food product [27]. Starch-based films developed by Basiak, Debeaufort and Lenart [28] had a water swelling value of 40% when placed in distilled water for 2 min. High swelling values in water at the level of 126 ± 1–500 ± 13% were also obtained by Pająk, Przetaczek-Rożnowska and Juszczak [18]. Those authors tested starch-based films but placed the films in distilled water for 24 h. Moreover, those authors observed a similar relationship because the films after this treatment were not damaged, unlike the films made on the basis of oilseed flours. The high water resistance of edible films depends on the ratio of amylose to amylopectin present in the polymers. Further studies are recommended to better define the interactions between amylose, amylopectin, glycerol and water and biopolymers. As a result, it will be possible to better determine their influence on the properties of edible films.

#### 2.2.5. Film Opacity

The opacity of the edible films is a very important parameter as it determines the visibility of the packaged food products to consumers. Opacity also determines the ability of materials to refract light, and the obtained results indicate greater refraction of light, resulting from the presence of flours in the film-making material (Table 2).

According to Acquah, Zhang, Dube and Udenigwe [29], high film opacity values are associated with the presence of phenolic compounds in the samples tested. However, according to Zheng, Yu and Pilla [30], good barrier properties against UV radiation are due to the presence of amino acid residues in proteins that have the ability to absorb UV radiation. The opacity value of the edible films obtained from bocaiuva flour ranged from 3.8 to 9.3 A/mm [31]. Films obtained on the basis of peas by Giosafatto et al. [2] had an opacity value of 7.74 ± 0.26 at a wavelength of 600 nm. The result obtained was also similar to the bean (Phaseolus vulgaris) and pea (Pisum sativum) protein hydrocolloid films obtained by Shevkani and Singh [32]. The film opacity for the soy films obtained by Ortiz et al. [33] was 1.00 ± 0.05 for films dried at 50 °C and 1.50 ± 0.05 for films dried at 40 °C.

#### 2.2.6. Color

The color of edible films is a very important parameter influencing the acceptance of products by consumers; therefore, transparent, bright and almost invisible films are mostly expected. The color of the films obtained varied depending on the type of flour used. The control films were characterized by high brightness and transparency, while the films containing added flour were opaque. The color values of the obtained edible films are presented in Table 2. The results show that the lowest brightness, 34.91 (parameter L*), and the darkest color were found for the evening primrose flour film. The highest value of the L* brightness parameter was obtained for the edible film from the SPI soy protein isolate (91.84). Films made from banana peel flour by de Faria Arquelau et al. [34] had a yellow color, and the obtained L* value was 82.47. It was also found that the value of the brightness parameter was influenced by the heating time. The shorter the heating time, the greater the brightness of the edible film. The a* color parameter of the obtained edible films ranged from −5.13 for the sunflower flour film to 13.62 for the linseed flour film. Positive values of the a* parameter indicate a greater proportion of red, while negative values indicate the presence of a greater proportion of green. The highest proportion of green color was observed for the film made of sunflower flour, the film-forming solution of which, after adding NaOH solution, changed the color from light brown to green. The differences in the colors of the edible films depended on the type of flour additive used and were characterized by significant differences (*p* < 0.05). Additionally, the addition of flour resulted in a significant deviation from the pattern of the soy protein isolate film and an increase in the amount of dyes present (ΔE). The obtained values of the absolute color difference (ΔE) in the range 27.47–64.82 showed a significant deviation from the standard. According to the criterion adopted by the International Commission on Lighting, the values in the range 0–2 are unrecognizable for humans. An inexperienced observer will recognize color deviation differences in the range of 2–3.5, while clear differences are visible at values higher than 3.5 [35]. Maniglia, Tessaro, Lucas and Tapia-Blácido [36] also obtained high values of the absolute color difference for babassu mesocarp flour films in the range 22.46 ± 0.89–41.77 ± 0.48.

### 2.3. Water Vapor Permeability, Sorption and Diffusion

One of the main purposes of using edible films is to control the possibility of water vapor migration between the coated food product and the surrounding atmosphere. Therefore, the aim is to achieve a relatively low water vapor permeability. The control film from soy protein isolate had the most compact structure and the lowest water vapor permeability parameters, while the films containing added flour had higher values (Table 3). It was observed that films of pumpkin seed flour and sesame flour, which contained the highest amount of protein in their composition, were characterized by the highest water vapor permeability value. According to [10], the presence of proteins and soluble fibers in flour increases the number of interactions between flour components and water molecules, which increases the water vapor permeability of the film. Wu et al. [37] obtained water vapor permeability values for pomelo flour films in the range 2.02 ± 0.02–2.95 ± 0.07 × 10^−12^ g cm cm^−2^ s^−1^ Pa^−1^. According to those authors, a greater water vapor barrier results from the formation of denser composite film systems. On the other hand, the permeability for the films of achira flour was 5.3 ± 0.2 × 10^−10^ g m^−1^ s^−1^ Pa^−1^ [20], for films based on quinoa, it was 0.6 ± 0.1 × 10^−10^ g m^−1^ s^−1^ Pa^−1^ [22] and for banana flour films, it was 2.1 ± 0.2 × 10^−10^ g m^−1^ s^−1^ Pa^−1^ [38].

**Figure 2:**
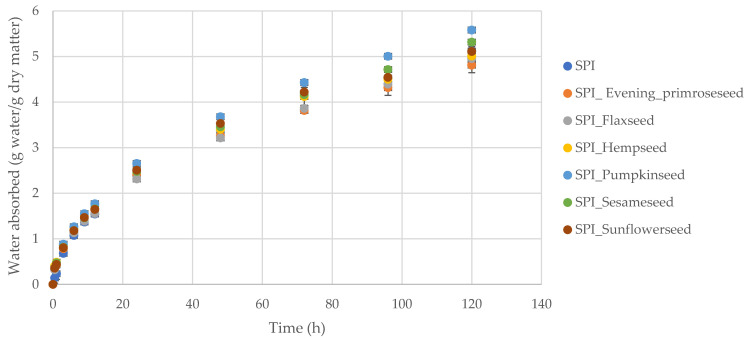
Kinetics of water vapor adsorption by soy protein isolate–oilseed flour films.

The shape of the curves of the dependence of the amount of adsorbed water on time is, in all cases, characterized by a similar shape (Figure 2). The highest increase in water content was recorded for the film made of pumpkin seed flour, while the lowest value and thus the highest surface hydrophobicity were found for the film made of evening primrose flour. It was observed that in the first hours of the process, there was the fastest increase, while in the following hours, it stabilized, but without reaching equilibrium due to hygroscopicity. According to Tapia-Blácido, do Amaral Sobral and Menegalli [38], edible films of amaranth flour that are plasticized with glycerol rather than sorbitol have an improved equilibrium water-holding capacity. Films with glycerol, even at high temperatures, have been found to be more hygroscopic than those plasticized with sorbitol. Due to the greater affinity of glycerol to water, it is possible to obtain a better plasticizing effect.

Table 3 shows the water vapor diffusion coefficient values for soybean films with oilseed flour added. It was observed that the addition of flours caused an increase in the value of the water vapor diffusion coefficient, which could have been caused by an increase in the porosity of the films resulting from the addition of flours, as well as different chemical compositions. Dias, Müller, Larotonda and Laurindo [11], for films based on rice flour, obtained values of the water vapor diffusion coefficient in the range 1.2–6.6 × 10^−13^ m^2^ s^−1^. On the other hand, Zhao et al. [39], for films with the addition of rice flour, obtained values of the water vapor diffusion coefficient in the range from 3.06 × 10^−14^ to 2.48 × 10^−15^ m^2^ s^−1^.

### 2.4. Microstructure

**Figure 3:**
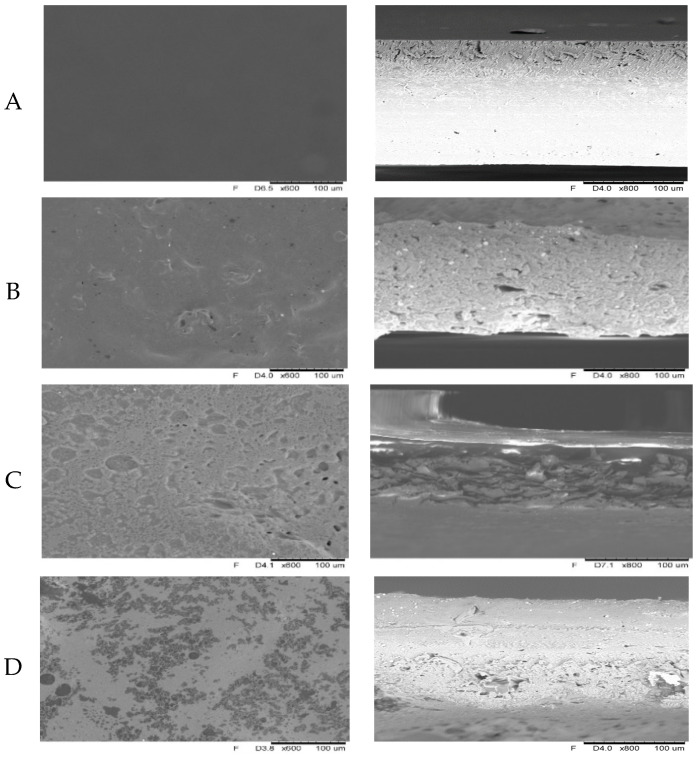

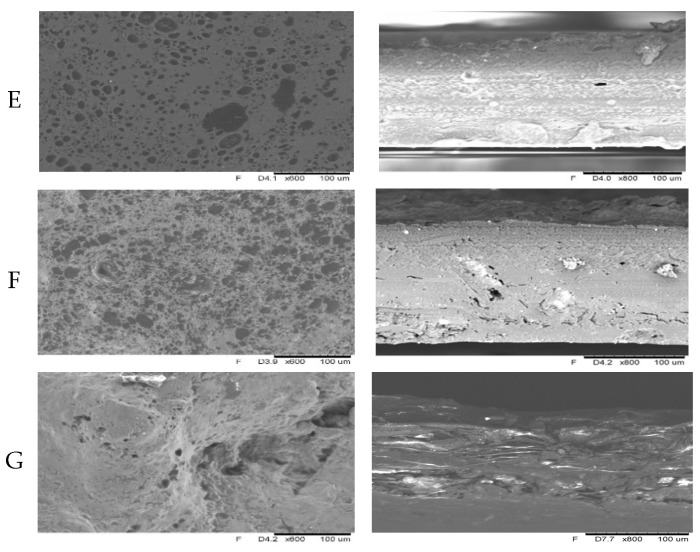
Film micrographs of surfaces and cross-sections of soy protein isolate–oilseed flour films (**A**—SPI, **B**—SPI_Flaxseed, **C**—SPI_Hempseed, **D**—SPI_Sesameseed, **E**—SPI_Sunflowerseed, **F**—SPI_ Evening_primroseseed, **G**—SPI_Pumpkinseed).

Figure 3 shows the scanning microscope photographs of the surfaces and sections of the analyzed films. The control films from the soy protein isolate had a homogeneous and smooth surface, while the films made with oilseed flours were heterogeneous and rough. On the surface of the tested films, there were irregularly arranged fat drops, which differed in size and shape. Irregular cracks are visible in the micrograph of the surface of films made of pumpkin seed flour. The highest surface roughness was observed in the case of films made of evening primrose flour. According to Gutiérrez and González [40], the rougher the coating, the more opaque the film. The consistent morphology of the edible films makes it possible to reduce water adsorption and film polarity. However, according to Drakos et al. [41], the differences resulting from the consistency of the films could be due to the different contents of protein and carbohydrates in the flours used. Additionally, Silva, Cortez-Vega, Prentice and Fonseca [31], analyzing the obtained edible films from bocaiuva flour, observed a porous structure and unevenness of the film surface, which could interfere with water vapor diffusion. Those researchers also observed the presence of lipid globules separated from the starch matrix found in the darker regions of the films.

### 2.5. Mechanical Properties

**Figure 4:**
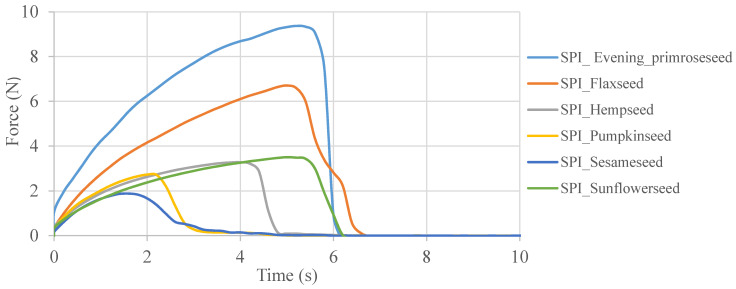
Force–time curves occurring during the tensile strength analysis of soy protein isolate–oilseed flour films.

The restriction of the use of edible films is often due to poor mechanical properties compared to packaging made of synthetic polymers [42]. The tensile strength, elongation and Young’s modulus values are given in Table 4. Figure 4 presents the curves showing the relationship between the force required to break the sample and time. The curves showing the stretching dependence of edible films obtained from evening primrose, flaxseed and sunflower flours are characterized by the greatest stretch. Edible films obtained from sesame flour and pumpkin seed flour turned out to be the least stretchy. Generally, all films with the addition of oilseed flour were characterized by low values of mechanical strength. However, the edible films obtained on the basis of evening primrose flour, characterized by evenly distributed fat droplets (Figure 3), with a fairly regular shape, achieved the highest tensile strength. Moreover, the film based on evening primrose flour was also characterized by the highest thickness value (173.50 ± 15.21) (Table 1).

After comparing the properties of the films, significant differences were observed. The tensile strength values ranged from 0.59 to 3.09 MPa. Considering the use of the same amount of glycerol in the preparation of film-forming solutions in order to facilitate processing, it can be concluded that the differences in mechanical properties depended only on the types of biopolymers and their structure. The differences in mechanical properties might also be influenced by differences in thickness, as well as different water contents in the obtained films. According to the standards, in order for edible films to be used as biodegradable packaging materials, they should have a tensile strength above 3.5 MPa. The addition of a plasticizer, which was glycerol, caused a decrease in the affinity between the starch chains in the starch matrix. As a result, the formation of hydrogen bonds between the plasticizer and starch resulted in greater flexibility of the film [19]. The best mechanical properties were demonstrated by the film based on evening primrose flour, which contained the most carbohydrates in its composition compared to other flours. The high tensile value shows a homogeneous flour dispersion in the resulting edible film. High tensile strength values were obtained by Andrade-Pizarro, Skurtys and Osorio-Lira [13] during the production of gelatin films from cellulose nanofibers (23.50 and 52.72 MPa). Taking into account the parameter of the relative elongation of the films, it was observed that the control film had the lowest value (3.95%). After the research, it was found that the addition of flour increases the elongation of the edible films. Moreover, it was observed that for all the samples prepared with the addition of oilseed flour, the tensile strength increased as the elongation of the sample decreased. The higher the Young’s modulus value, the greater the stiffness of the coating material. Ramadhani, Rostini, Anna and Rochima [43] obtained values of elongation of flour films from seaweed plasticized with glycerol in the range of 18.3–86.7%. The value of Young’s modulus for the obtained coatings ranged from 1.19 ± 0.37 MPa for the control film to 18.75 ± 4.40 MPa for the evening primrose flour film. Young’s modulus values in the range 4.9 ± 0.4–9.3 ± 0.7 MPa were observed by Gutiérrez, Herniou-Julien, Álvarez and Álvarez [44] when obtaining films from guinea starch arrowroot. Edible films prepared from soy protein and galactomannan, the fraction extracted from the seeds of *Gleditsia triacanthos* (*Fabaceae*), were characterized by tensile strengths of 2.58 ± 0.08 MPa and 3.72 ± 0.04 MPa depending on the proportions used. Additionally, these edible films had a greater elongation at break of 27.4 ± 2.4% and 38.0 ± 3.0% [45].

### 2.6. Thermal Properties

**Figure 5:**
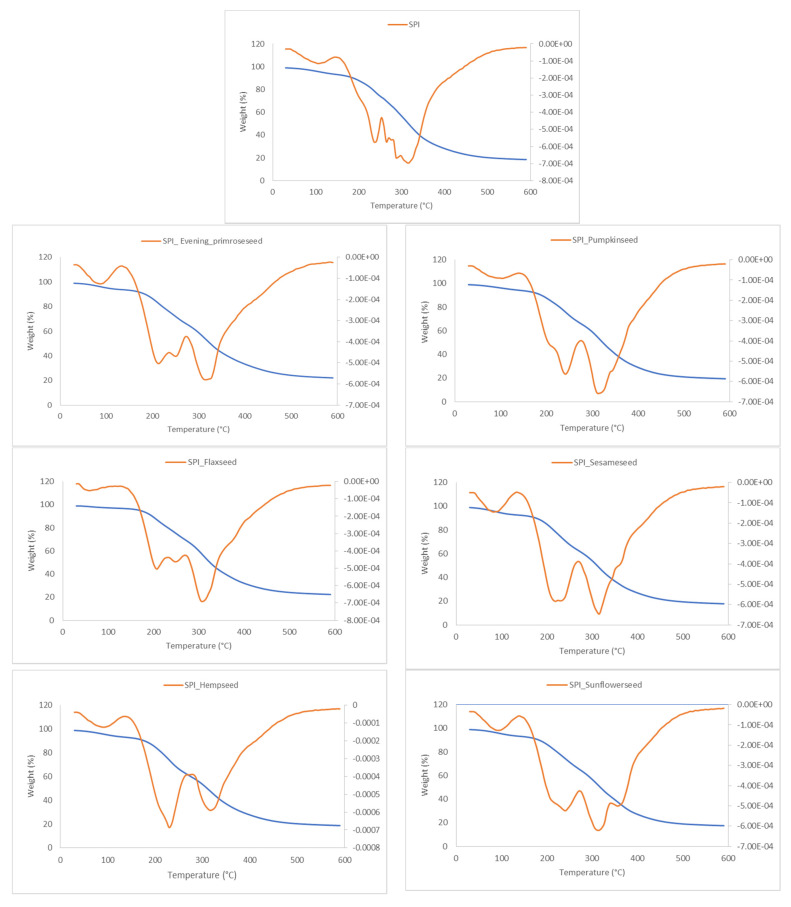
Thermogravimetric analysis (TGA) (blue) and derivative thermogravimetry (dTG) (orange) curves of films of soy protein isolate and oilseed flour.

The thermogravimetric analysis (TGA) curves and their first derivatives (dTG) are presented in Figure 5 and were investigated to assess the thermal stability of the films (Table 5). The dTG curves were shifted vertically for easier comparison. It can be observed that all analyzed films showed a similar behavior with three main stages of weight loss (Figure 5). The first stage was observed up to 100 °C and was related to the loss of the adsorber and bound water.

Usually, the first stage, from 25 to 200 °C, is attributed to the evaporation of water and molecules with a molecular weight [46]. The film composition affected this stage. Soy protein films without oilseed flours showed the highest temperature (99.98 °C), whereas films containing flaxseed flour showed the lowest temperature (53.20 °C) and lowest weight loss (3.47%), which is connected with a lower thermal stability. Other films exhibited weight loss from 5.88 to 7.65% at the range of temperature from 80.12 to 86.79 °C. Similar results, were reported by Tongnuanchan et al. [47] for fish gelatin films incorporated with palm oil. The second stage of the degradation temperature appeared approximately between 205.1 and 266.2 °C for films containing flaxseed flour and soy films without oilseed flours, respectively.

Soy films showed the highest temperature, and the addition of flours decreased this initial stage, resulting in lower stability, which may be due to the decomposition of protein fractions of a lower molecular weight. Regarding weight loss, it can be observed that at the second stage, the lowest value was observed for soy films (7.41%) and highest for films containing sunflower seed flour (28.49%) and sesame seed flour (29.81%). In general, the second stage, from 200 to 350 °C, is attributed to the thermal decomposition of the components in the analyzed films. According to the previous works, protein breakdown starts at the temperature of 225 °C [48], and the decomposition of starch is related to the temperature from 230 to 326 °C [49]. In the third stage, all films presented similar minor degradation temperatures, between 310.7 and 320.7 °C. However, the weight loss was at 20.96% for soy films and varied for other films between 12.83 and 14.53% for films with evening primrose seed, flaxseed and hemp seed flours, and between 25.41 and 37.44% for films containing sesame seed and sunflower seed flours, respectively. In general, in the third stage, above 350 °C, the degradation of carbonaceous residues which were formed during the second stage can be observed, combined with the complex oxidation of these materials [50]. Therefore, the thermogravimetric analysis demonstrated that the blending of soy protein with oilseed flours decreased the stability of soy films observed by the lower heat resistance and intra/intermolecular protein interactions of the analyzed films. This was better observed for the first and second stages when degradation occurred at lower temperatures. This can be attributed to the film composition, especially the lipid content, which was different for oilseed flours, and was from 6% for hemp flour to 16% for pumpkin seed flour, as well as the protein content, from 29% for evening primrose seed flour to 58% for pumpkin seed flour. In addition, the lower stability of the blended films might also be due to the destruction of hydrogen bonds between the protein and other molecules.

## 3. Materials and Methods

### 3.1. Materials

Soy protein isolate (SUPRO 670, ~95 g protein) was purchased from The Solae Company (DuPont, Warsaw, Poland). Oilseed flours (evening primrose, flax, hemp, pumpkin, sesame and sunflower) were produced by PPHU Machines and Processing of Oil Seeds Ol’Vita Krzysztof Dziaduch (Pszenno, Poland) and The Helcom Company (Helcom, Greek Trade Sp. z o.o., Kraków, Poland). Anhydrous glycerol, sodium hydroxide and sodium chloride were purchased from Avantor Performance Materials Poland S.A. (Gliwice, Poland).

#### 3.1.1. Characterization of Oilseed Flours

The oilseed flours’ particle size distribution was determined by sieve analysis with a vibratory sieve shaker model AS 200 (Retsch, Katowice, Poland) using a sieve aperture of 150, 150, 355, 500 and 710 μm for 10 min of sieving time. Table 6 shows the basic chemical composition of oilseed flours that were used to obtain edible films.

#### 3.1.2. Film Preparation

Films were prepared according to the casting method. Aqueous film-forming solutions were prepared from a mixture of oilseed flour and soy protein isolate at the concentration of 5% (*w*/*w*) and were mixed at 70 ± 1 °C for 20 min under 250 rpm with a constant magnetic stirrer, model RTC basic IKAMAG (IKA-Werke GmbH & Co., Staufen, Germany). Then, solutions were cooled down to 25 ± 1 °C, and glycerol (plasticizer) at 50% (*w*/*w*) was added. Control film-forming aqueous solutions were prepared without oilseed flours at the concentration of 10% (*w*/*w*) of soy protein isolate and with the addition of glycerol (50%). The pH of the prepared film-forming solutions using a pH meter (CPO-505, Warsaw, Poland) was adjusted to 10.0 with 1 M NaOH (sodium hydroxide) solution. The obtained film-forming solutions were poured into Petri dishes with a diameter of 10 cm in the amount of 10 mL.

**Figure 6:**
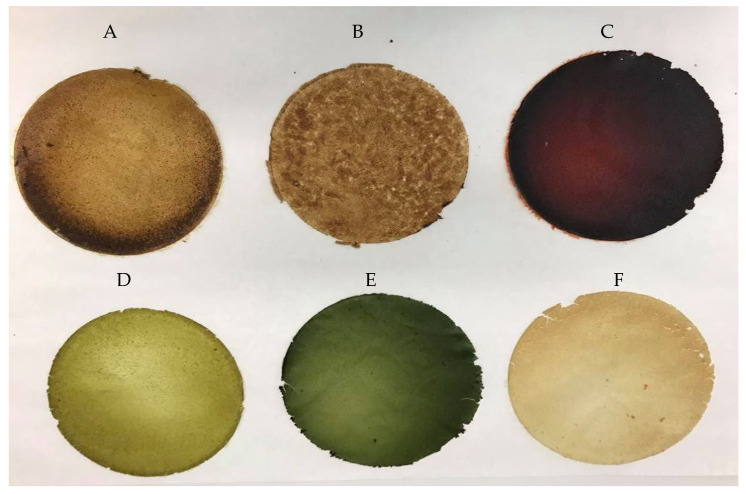
Edible films based on soy protein isolate and oilseed flours (**A**—SPI_Hempseed; **B**—SPI_Flaxseed; **C**—SPI_ Evening_primroseseed; **D**—SPI_Pumpkinseed; **E**—SPI_Sunflowerseed; **F**—SPI_Sesameseed).

The films were dried in a thermostatic chamber, model KBF 720 (Binder, Tuttlingen, Germany), at 25 °C and relative humidity of 50% for 24 h. Then, all films were removed from the Petri dishes and were conditioned for 48 h at 25 °C and relative humidity of 50% prior to testing (Figure 6).

### 3.2. Physical Properties

#### 3.2.1. Water Content

The water content was determined by the drying method at 105 °C for 24 h (dryer SUP 65 W/G, WAMED, Warsaw, Poland). Then, the percentage of dry residue present in the sample was calculated. The measurement was performed in triplicate.

#### 3.2.2. Thickness

The thickness of films was determined using an electronic gauge Ultrameter A400 (Metrison, Mościska, Poland) with the precision of 1 μm. The results were obtained by measuring the thickness at ten random points, and then the mean and the standard deviation were calculated.

#### 3.2.3. Water Solubility

The prepared samples of edible films were cut into squares (20 × 20 mm). The samples were then dried at 105 °C for 24 h. After this time, the samples were cooled in a desiccator containing silica gel. The samples were reweighed and placed in 25 mL of distilled water. After 24 h of storage, excess water was removed with filter paper. The samples were dried for 24 h at 105 °C and reweighed. The water solubility of the edible films was determined in three repetitions and was calculated based on the method described by Rhim, Lee and Ng [51].

#### 3.2.4. Swelling Index

This parameter was determined according to the method of Cao, Fu and He [52]. The film specimen was cut into square pieces (20 × 20 mm), and a piece of the edible film was accurately weighted. The film sample was placed in a glass beaker containing 25 mL of distilled water for 2 min. Then, all samples were drained and weighed, and the swelling index was calculated as the percentage of swelled water. The measurement was performed in three repetitions.

#### 3.2.5. Film Opacity

Opacity was determined according to the spectrophotometric method using the UV/VIS Helios Gamma spectrophotometer (Thermo Electron Corporation, Bath, UK). The absorbance was measured at 600 nm on 100 × 400 mm samples in ten repetitions, and an empty test cell was used as reference. The opacity of the prepared material was calculated according to the method described by Han and Floros [53] and expressed as absorbance per film thickness in mm.

#### 3.2.6. Color

The color test was performed using the CIE L*a*b* system (L*—brightness, a*—green to red color, b*—blue to yellow color) using the CR-300 model colorimeter (Minolta, Tokyo, Japan). The measurement was performed in ten repetitions. For a better interpretation, the formula for the total color difference (∆E) between the film and the white standard (L* = 99.27 ± 0.10; a* = 0.019 ± 0.06; b* = 1.23 ± 0.19) was calculated according to the method described by Sobral, dos Santos and Garcia [54].

### 3.3. Water Vapor Permeability (WVP), Sorption and Diffusion

The water vapor permeability of the obtained films was determined by the gravimetric method (Debeaufort, Martin-Polo and Voilley, 1993) [55]. An RH difference of (50–100%) at a temperature of 25 ± 1 °C was used. At least three replicates were performed for each film type, and the WVP was determined at steady state and from the change in the cell mass as a function of time.

The water vapor sorption kinetics were measured after 0.5, 1, 3, 6, 9, 12, 24, 48, 72, 96 and 120 h under constant temperature of 25 °C and relative humidity of 75.3%. For each type of film, at least 3 repetitions were performed. The kinetic curves were plotted as graphs showing the dependence of the change in the amount of adsorbed water on the time of the process.

The water vapor diffusion coefficient was estimated by approximation and valid for the used testing conditions only according to the method described by Galus et al. [56] based on Fick’s second law (Crank, 1975) [57].

### 3.4. Microstructure

The films were previously cut into small squares of 5 × 5 mm and fixed on a metallic cylindrical support. Film surfaces and film cross-sections were analyzed by scanning electron microscopy (FEI Company, Quanta 200 MK2, Fremont, CA, USA) at a magnification of x600 (surfaces) and x800 (cross-sections).

### 3.5. Mechanical Properties

The ASTM standard method D882-02 (ASTM, 2002) was used to determine tensile strength (TS), elongation at break (E) and Young’s modulus (YM) of the analyzed films. The Texture Analyzer TA-XT2i (Stable Micro Systems, Haslemere, UK) with the Texture Expert software was used to process the results. Measurements of the mechanical properties of the edible films were performed in at least six repetitions.

### 3.6. Thermal Properties

Thermogravimetric analyses were performed using a TGA thermal analyzer (Mettler Toledo, Warszawa, Poland) to determine thermal stability and degradation of the films. Each film sample (5 mg) was heated at 5 °C min^−1^ from 30 to 600 °C under nitrogen atmosphere (N_2_ flow was 50 mL min^−1^). TGA and DTG curves were acquired from the differential TGA values.

### 3.7. Statistical Analysis

The results were evaluated by one-way analysis of variance (ANOVA) using the software Statistica 13.0 (StatSoft Inc., Kraków, Poland). Tukey’s post hoc test was performed to compare the results, expressed by mean (±) standard deviation, at the level of significance of 0.05.

## 4. Conclusions

It was found that the addition of oilseed flour is a good base material for the preparation of composite edible films. The type of flour used influenced the differentiation of the film properties as well as their functionality. The addition of flours shows that the use of the correct type of flour makes it possible to improve the effectiveness of the film used as a moisture barrier or its adsorptive properties. The results obtained in this study suggest that the oilseed flours added to film-forming solutions are promising materials that can be used in the development of biodegradable films and applied in the food industry for packaging food products. The best mechanical properties and the greatest durability were achieved in the preparation of evening primrose flour films. From the practical point of view, the analyzed soy protein–oilseed flour films can be applied as edible protective coatings for different products, including vegetable bars, burgers or other processed foods based on vegetables or seeds.

## Figures and Tables

**Table 1:** Water content, thickness and water solubility of soy protein isolate–oilseed flour films.

Film	Water Content (%)	Thickness (μm)	Water Solubility (%)	Swelling in Water (%)
SPI	6.72 ± 0.07 ^ab^	120.50 ± 12.50 ^b^	100%	n.d.
SPI_ Evening_primroseseed	6.00 ± 0.55 ^a^	173.50 ± 15.21 ^a^	14.39 ± 0.63 ^abc^	191.78 ± 4.93 ^bc^
SPI_Flaxseed	8.50 ± 0.78 ^c^	164.89 ± 12.97 ^a^	12.18 ± 2.04 ^ab^	362.16 ± 4.01 ^d^
SPI_Hempseed	6.88 ± 0.31 ^ab^	164.78 ± 16.69 ^a^	15.76 ± 1.41 ^bc^	170.43 ± 16.23 ^b^
SPI_Pumpkinseed	6.50 ± 0.53 ^ab^	155.88 ± 18.04 ^a^	11.38 ± 2.33 ^a^	131.10 ± 1.86 ^a^
SPI_Sesameseed	7.92 ± 0.48 ^bc^	167.57 ± 16.20 ^a^	17.25 ± 1.41 ^c^	139.78 ± 5.52 ^a^
SPI_Sunflowerseed	8.45 ± 0.56 ^c^	137.60 ± 14.25 ^b^	13.80 ± 1.65 ^abc^	206.58 ± 8.68 ^c^

Mean values with standard deviations in brackets. Different superscript letters (^a–d^) within the same column indicate significant differences between the films (*p* < 0.05) (n.d.—not determined).

**Table 2:** Color attributes (L*, a*, b*) and total color difference (ΔE) of soy protein isolate–oilseed flour films.

Film	L*	a*	b*	ΔE	Film Opacity (A mm^−1^)
SPI	91.84 ± 1.05 ^f^	−3.18 ± 0.21 ^b^	25.72 ± 2.39 ^b^	92.04 ± 1.51 ^f^	1.27 ± 0.28 ^c^
SPI_ Evening_primroseseed	34.91 ± 1.13 ^b^	6.99 ± 0.71 ^d^	1.35 ± 0.66 ^c^	64.82 ± 0.99 ^a^	9.57 ± 0.74 ^e^
SPI_Flaxseed	54.28 ± 3.03 ^a^	13.62 ± 0.96 ^e^	21.06 ± 2.11 ^a^	65.42 ± 2.47 ^a^	7.15 ± 0.96 ^b^
SPI_Hempseed	63.16 ± 3.18 ^c^	8.66 ± 0.90 ^d^	30.24 ± 1.33 ^d^	47.17 ± 2.04 ^c^	3.85 ± 0.64 ^d^
SPI_Pumpkinseed	71.46 ± 1.56 ^d^	−1.80 ± 0.73 ^b^	34.40 ± 0.39 ^e^	71.69 ± 1.36 ^e^	5.59 ± 1.22 ^a^
SPI_Sesameseed	83.40 ± 1.08 ^e^	1.15 ± 0.37 ^c^	25.21 ± 1.05 ^b^	28.74 ± 1.34 ^b^	6.12 ± 0.56 ^a^
SPI_Sunflowerseed	51.97 ± 2.85 ^a^	−5.13 ± 0.99 ^a^	19.88 ± 2.23 ^a^	51.18 ± 1.83 ^d^	7.57 ± 0.67 ^b^

Mean values with standard deviations in brackets. Different superscript letters (^a–f^) within the same column indicate significant differences between the films (*p* < 0.05).

**Table 3:** Water vapor permeability (WVP) at relative humidity difference of 50–100% and water diffusion of soy protein isolate–oilseed flour films.

Film	WVP (10^−9^ g m^−1^ s^−1^ Pa^−1^)	Water Diffusion (10^−14^ m^2^ s^−1^)
SPI	1.49 ± 0.13 ^a^	0.80 ± 0.00 ^e^
SPI_ Evening_primroseseed	2.28 ± 0.33 ^e^	1.65 ± 0.07 ^c^
SPI_Flaxseed	1.82 ± 0.28 ^c^	1.40 ± 0.06 ^ab^
SPI_Hempseed	1.94 ± 0.17 ^d^	1.55 ± 0.07 ^bc^
SPI_Pumpkinseed	2.41 ± 0.27 ^f^	1.35 ± 0.07 ^a^
SPI_Sesameseed	2.70 ± 0.38 ^g^	1.50 ± 0.06 ^ab^
SPI_Sunflowerseed	1.64 ± 0.21 ^b^	1.15 ± 0.07 ^d^

Mean values with standard deviations in brackets. Different superscript letters (^a–g^) within the same column indicate significant differences between the films (*p* < 0.05).

**Table 4:** Mechanical properties of films prepared with oilseed flours.

Film	Tensile Strength (MPa)	Young’s Modulus (MPa)	Elongation at Break (%)
SPI	1.74 ± 0.23 ^c^	1.19 ± 0.37 ^a^	3.95 ± 0.15 ^c^
SPI_ Evening_primroseseed	3.09 ± 0.34 ^d^	18.75 ± 4.40 ^d^	24.54 ± 4.75 ^b^
SPI_Flaxseed	2.04 ± 0.36 ^c^	10.20 ± 2.33 ^c^	20.03 ± 3.37 ^ab^
SPI_Hempseed	1.21 ± 0.19 ^b^	5.12 ± 1.72 ^b^	16.57 ± 3.87 ^a^
SPI_Pumpkinseed	0.82 ± 0.23 ^a^	1.85 ± 0.94 ^a^	8.49 ± 2.46 ^d^
SPI_Sesameseed	0.60 ± 0.24 ^a^	1.01 ± 0.72 ^a^	6.04 ± 2.60 ^cd^
SPI_Sunflowerseed	1.28 ± 0.15 ^b^	6.36 ± 1.14 ^b^	19.77 ± 1.97 ^ab^

Mean values with standard deviations in brackets. Different superscript letters (^a–d^) within the same column indicate significant differences between the films (*p* < 0.05).

**Table 5:** Temperature and weight loss related to stages of TG/dTG curves of soy protein isolate–oilseed flour films.

Film	First Stage		Second Stage		Third Stage	
	Temperature (°C)	Weight Loss (%)	Temperature (°C)	Weight Loss (%)	Temperature (°C)	Weight Loss (%)
SPI	99.98	7.06	266.2	7.41	320.7	20.96
SPI_ Evening_primroseseed	82.09	6.25	210.6	17.88	318.4	13.93
SPI_Flaxseed	53.20	3.47	205.1	15.84	310.7	13.07
SPI_Hempseed	85.87	6.85	230.6	19.75	320.33	12.83
SPI_Pumpkinseed	86.79	5.88	242.4	18.13	316.8	14.53
SPI_Sesameseed	80.12	7.65	218.6	29.81	316.9	35.41
SPI_Sunflowerseed	86.38	7.04	242.2	28.49	318.3	37.44

**Table 6:** Chemical composition of selected oilseed flours (in 100 g of the product).

	Evening Primroseed	Flaxseed	Hempseed	Pumpkinseed	Sesameseed	Sunflowerseed
Energy (kcal)	363.8	316	356	425.2	397.5	346
Fat (g)	8	8.9	6	16	10	9.5
of which saturates (g)	1	0.8	1	3	2	1.2
Carbohydrate (g)	45	4.8	41	13	21	8
of which sugars (g)	1	4.1	3	1	1	6.5
Protein (g)	29	38	38	58	55	48
Salt (g)	0	0.16	0	0	0	0.01

## Data Availability

Data sharing not applicable.

## Sample Availability

Samples of the films are available from the authors.
